# Porous titanium microsphere kyphoplasty for augmentation treatment of osteoporotic vertebral fractures: Technical report and case series

**DOI:** 10.3389/fsurg.2023.1152995

**Published:** 2023-05-03

**Authors:** Fulvio Tartara, Daniele Armocida, Diego Garbossa, Francesco Meli, Gabriele Costantino, Fabio Cofano, Natale Francaviglia

**Affiliations:** ^1^ Headache Science and Neurorehabilitation Center, IRCCS Mondino Foundation, Pavia, Italy; ^2^ Department of Brain and Behavioral Sciences, University of Pavia, Pavia, Italy; ^3^ Neurosurgery Division, Human Neurosciences Department, “Sapienza” University, AOU Policlinico Umberto I, Rome, Italy; ^4^ IRCCS Neuromed, Pozzilli, Italy; ^5^ Neurosurgery Division, Department of Neuroscience, A.O.U. Città della Salute e della Scienza, University of Turin, Turin, Italy; ^6^ Neurosurgery Division, Arnas Civico di Cristina, Palermo, Italy

**Keywords:** vertebroplasty, kyphoplasty, poly (methyl methacrylate) (PMMA), titanium-based composite, osteoporotic age-related fractures

## Abstract

**Background:**

Vertebral augmentation procedures (VAPs) are used in cases of persistent and unresponsive pain in patients with vertebral compression fractures (VCFs). Although VAPs are considered a safe procedure providing quick pain relief and improved physical function, some postoperative complications can occur, for example, bone cement leakage. The material used in this procedure is almost exclusively polymethyl methacrylate (PMMA), which appears to lack biological activity and osteointegration capabilities. In this study, we introduce a new filling system consisting of cannulas preloaded with titanium microspheres, which stabilizes and consolidates the structure of the vertebral body in treating VCFs after the performance of the kyphoplasty procedure.

**Methods:**

We report a retrospective case series of six patients affected by osteoporotic vertebral fractures with worsening back pain, neurologic impairment, and failed conservative treatment who underwent the VAP at our institute, for which the SPHEROPLAST [MT ORTHO s.r.l., Aci Sant’Antonio (CT), Italy] system was used.

**Results:**

The patients had failed an average conservative trial of 3.9 weeks before they presented to us with neurodeficit. There were two men and four women with a mean age of 74.5 years. The average hospital stay was 2 days. There were no reported perioperative complications related to cement injection, such as intraoperative hypoxia, hypotension, pulmonary embolization, myocardial infarction, neurovascular or viscera injury, or death. The VAS score significantly decreased from a mean preoperative of 7.5 (range 6–19) to 3.8 (range 3–5) immediately after surgery and 1.8 (range 1–3).

**Conclusion:**

We report the first clinical results in a series of six patients treated for VCF using the microsphere system after analyzing the clinical results produced by, and the complications that arose from, this new device. In patients with VCF, the VAP using titanium microspheres appears to be a feasible and safe procedure with a low risk of material leakage.

## Introduction

Osteoporosis is the most frequently occurring bone disease with high incidence in a population of over 55 years of age. The occurrence of vertebral compression fractures (VCFs) is very common in patients with osteoporosis either in association with minor trauma or as a spontaneous event, whereas any sudden force of axial compression, distraction, or rotation on brittle bone could lead to osteoporotic fractures (1). Clinically, VCFs may be associated with invalidation of pain and disability, leading to significant limitations in the physical activity and quality of life of patients. In addition, fracture progression with progressive crushing and wedge deformation of the vertebral soma may contribute to the generation of segmental and overall kyphotic deformity of the spine. The initial treatment of VCFs is conservative, with the adoption of rigid orthoses and pain-drug therapy (2). Although conservative treatments can help manage most patients with osteoporotic vertebral fractures, debilitating pain and substantial functional limitations often continue to impact their quality of life (3). Vertebral augmentation procedures (VAPs) such as vertebroplasty and kyphoplasty are used in persistent and unresponsive cases of pain. These treatments consist of an injection into the vertebral body, through percutaneous needles (trokars), of material (cement) that can consolidate within the vertebra, stabilizing the fracture and imparting solidity to the vertebral soma itself. Although VAPs are considered a safe procedure providing quick pain relief and improved physical function, some postoperative complications can still occur, such as bone cement leakage, embolism, bone cement implantation syndrome, infection, thermal damage to surrounding soft tissue, and adjacent vertebral fracture (4, 5).

The material that is used almost exclusively in this procedure is polymethyl methacrylate (PMMA), which is available with different viscosities.

This type of material lacks complete biological activity and osteointegration capabilities. To date, only cases of bone cement displacement after the performance of the VAP have been reported. The factors closely related to the occurrence of bone cement displacement have not been determined. There are no systematic studies on bone cement displacement after the VAP, and a quantitative definition of bone cement displacement is lacking (5). In this preliminary study, we introduce a new augmentation system consisting of porous titanium microspheres that are able to stabilize and consolidate VCFs after a kyphoplasty procedure was performed. We report the first clinical results in a series of patients treated for VCF using the microsphere system after analyzing the clinical results produced by, and the complications arising from, this new device.

## Materials and methods

### Patient selection

The present study included patients with osteoporotic vertebral fractures and worsening back pain related to failed conservative treatment. These patients underwent the VAP at our institute between March and September 2022. In accordance with the World Health Organization’s (WHO) diagnostic criteria, osteoporosis in these patients was diagnosed with a T score of 2.5 by a dual-energy x-ray absorptiometry of the lumbar spine. The included patients had a bone mineral density (BMD) of more than 2.5 SD below the young adult mean (−2.5 SD or less). Patients younger than 65 years, patients with pathological fracture and osteomyelitis, and patients with a near-normal *T* score for BMD were excluded from the study. All patients provided personalized informed consent.

Clinical evaluation was done using the visual analog scale (VAS) for pain intensity, the severity of physical impairment was assessed using the Roland–Morris Low Back Pain and Disability Questionnaire (RMQ) impairment scale, and the severity of disability was assessed using the Oswestry Disability Index (ODI).

All patients had restrictions in standing and walking because of severe pain. For each patient, demographic data, the time between the clinical debut and the procedure, and potential complications were recorded. All patients who underwent the VAP were included for analysis regardless of surgical indication. Patients required a minimum of 3 months of follow-up for inclusion. This time point was chosen to optimize the number of patients evaluated for immediate postoperative complications.

All patients were evaluated by using plain radiographs, computed tomography scan (CT), and magnetic resonance imaging (MRI) to determine the morphology of the fracture.

**Figure 1:**
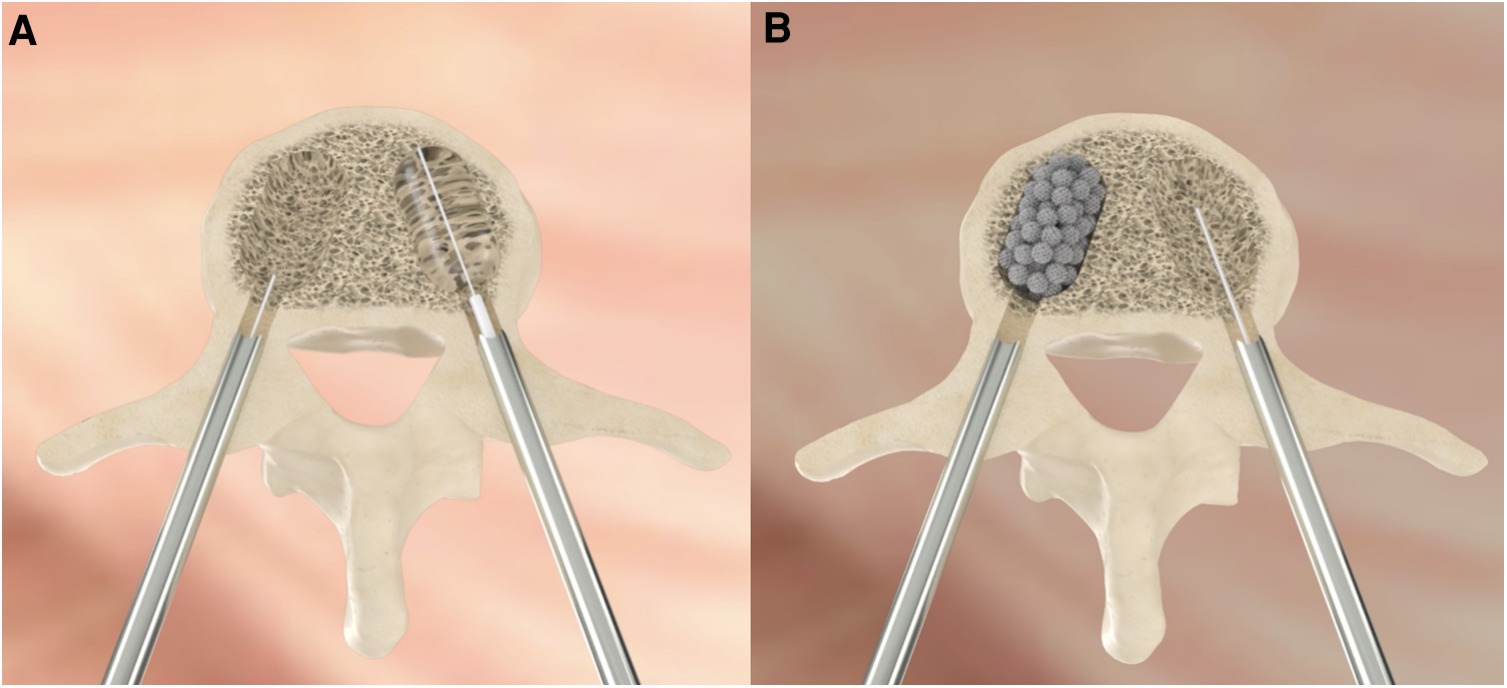
The figure shows the bilateral percutaneous approach to perform VAP introducing the ballon to restore the height (**A**) and the injection of the solution containing the titanium microspheres (**B**).

**Figure 2:**
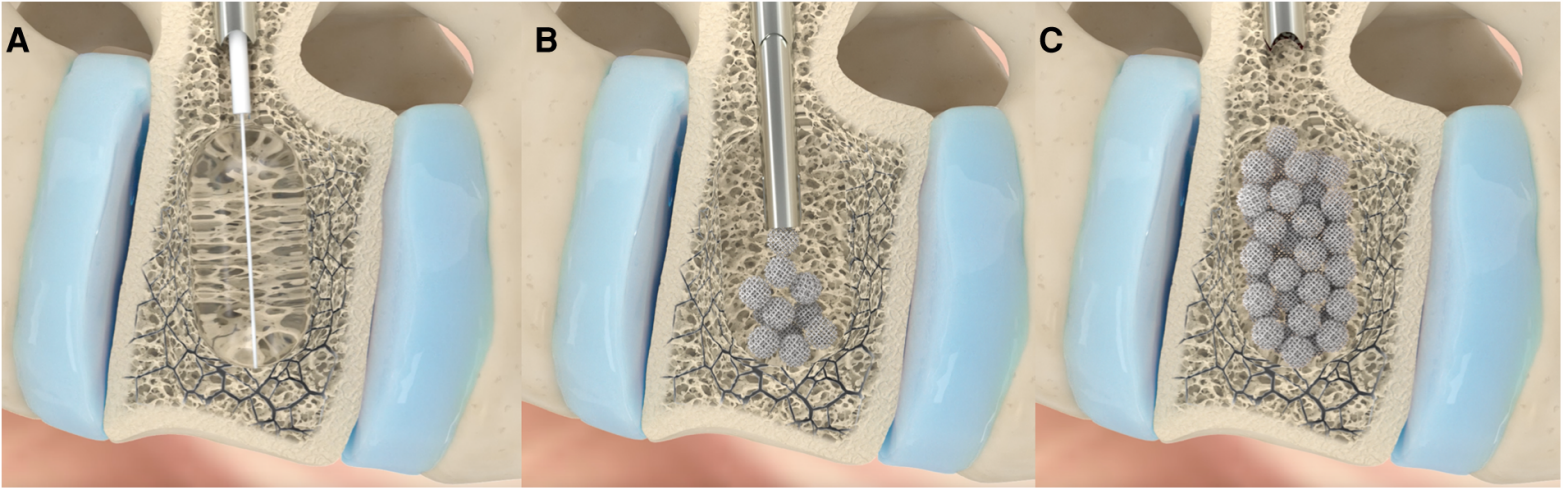
The main part of VAP treatment with spheroplast is schematized in this image. Through a percutaneous biportal access using trokar, the balloon is insufflated, restoring, as much as possible, the height of the fractured vertebra and creating a cavity (**A**). Then, the solution containing the titanium microspheres is injected in multiple jets; this step is performed under radioscopy (**B**). The injected material is then compacted until the cavity is fully occupied (**C**).

We used SPHEROPLAST [MT ORTHO s.r.l., Aci Sant’Antonio (CT), Italy], a filling system consisting of cannulas preloaded with spheres and a dedicated introducer with the aid of a compactor that was able to create a solid cast within the vertebral body for VCF augmentation. The surgical technique is illustrated in Figure 1. The patients were positioned prone on bolsters. This led to a partial correction of kyphosis and restoration of the anterior vertebral height (AVH) with the balloon compression (Figure 2). First, the pedicles of the affected segment were identified and the skin was marked. Trokar needles were passed into the pedicles together with a cutter to reach optimal position with image intensifier guidance. Then, 15 ml of balloon kyphoplasty were applied on both sides to create space for housing microspheres. Microspheres were then prepared and loaded into the introducer and progressively pushed through the needle. The procedure involves inserting the largest number of spheres to generate a solid cast covering the entire fracture surface. A compactor was used to facilitate microsphere progression.

An x-ray of the lateral and anterior views of the vertebrae was done during the microsphere introduction to observe the distribution pattern. The patients were mobilized after 3 h from the end of the procedure with braces. Clinical and radiological evaluations were done preoperatively, immediately postoperatively, and at 3 months after procedure.

## Results

### Patient demographics and clinical data

We report six patients who underwent the minimally invasive percutaneous technique VAP with spheroplasts included in the study. Electronic medical records of these patients were evaluated clinically, as shown in Table 1. These patients had failed an average conservative trial of 3.9 weeks (range 2–6 weeks) before they presented to us with invalidated worsening pain.

**Table 1:** Demographic data and clinical parameters of six patients.

No	Age	Sex	Level	Type	Time for procedure (days)	VAS pre	RMQ pre	ODI pre	Treatment	Deficit	Dislocation/leakage	Complications	VAS post	VAS FU	RMQ FU	ODI FU
1	66	M	L1	OF3	16	9	22	68	Biportal	No	No	No	5	1	6	18
2	71	F	L3	OF3	24	8	21	64	Biportal	No	No	No	4	2	5	16
3	76	F	D11	OF3	30	7	19	48	Biportal	No	No	No	4	3	8	10
4	72	F	L1	OF2	17	8	20	52	Biportal	No	No	No	4	2	7	14
5	82	F	L2	OF3	36	6	17	46	Biportal	No	No	No	3	1	5	8
6	80	M	L2	OF4	42	7	16	42	Biportal	No	No	No	3	2	5	10

FU, follow-up at last evaluation; Pre, preoperative evaluation; Post, postoperative evaluation at 1 day; OF, osteoporotic fracture classification of the Spine Section of the German Society for Orthopaedics and Trauma (DGOU).

There were two men and four women with a mean age of 74.5 years (range 66–82 years). The average hospital stay was 1.5 days (1–3 days).

**Figure 3:**
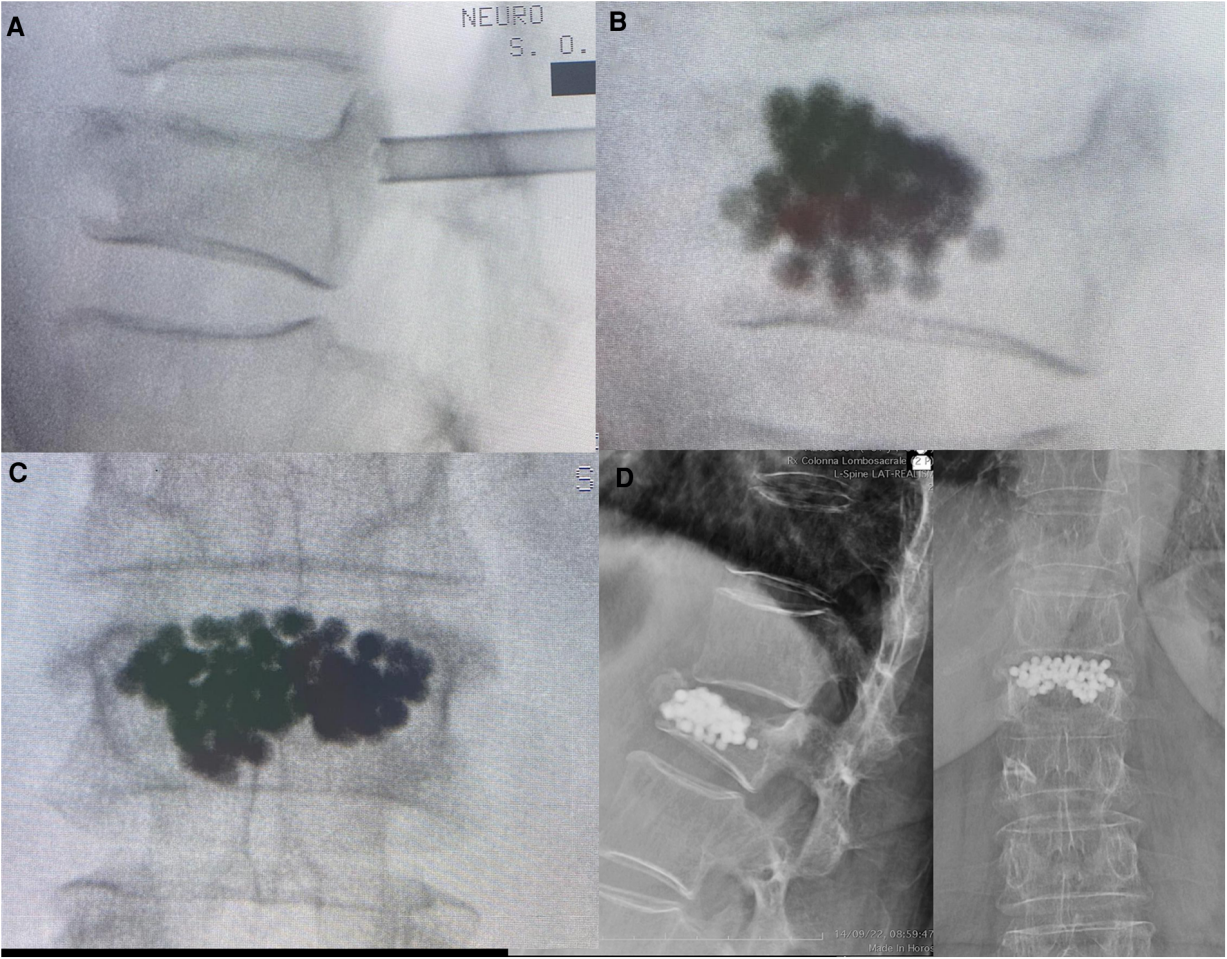
A representative case of an 82-year-old woman with vertebral osteoporotic compression L1 fracture without posterior wall involvement, unresponsive to pain drugs. (**A**) Cannula insertion and (**B,C**) intraoperative view after the titanium microsphere injection. (**D**) Radiographic follow-up control at 3 months with signs of partial ossification and fusion.

No perioperative complications related to microsphere injection were reported. In particular, a non-displacement of microspheres outside the vertebral body was observed. At 3-month follow-up, x-ray control revealed that the microsphere cast remained largely unchanged in its position with partial signs of fusion (Figure 3).

The VAS score significantly decreased from a mean preoperative 7.5 (range 6–9) to 3.8 (range 3–5) immediately after surgery and to 1.8 (range 1–3) at the 3-month follow-up. Statistical analysis could not be carried out because of the low number of patients involved in the study.

The RMQ and ODI scores decreased from a mean preoperative RMQ = 19.1 (range 16–22) and ODI = 53.3 (range 42–68) to RMQ = 6 (range 5–8) and ODI = 12.7 (range 8–18), respectively, at last evaluation at follow-up.

## Discussion

The VAP is a type of surgery that is used to restore the strength and stiffness of the vertebra after fractures caused by tumors, trauma, or osteoporosis. From the time this technique was introduced in 1987 by Galibert et al. (6), it has been used extensively to treat several traumas and diseases, particularly VCFs.

The VAP includes two kinds of surgeries: percutaneous vertebroplasty and percutaneous kyphoplasty. The core purpose of both is to insert bone cement into the fractured vertebral body (7). The treatment present optimal results only when it was used for “the most suitable patients” (8). VAPs have shown good uptake, and subsequent studies have shown the effectiveness of vertebroplasty procedures in controlling pain secondary to fracture conversely (9). Equally, a trend of kyphoplasty procedures scoring over vertebroplasty procedures has emerged (10). These methods have been widely used and remain viable in treating refractory pain secondary to osteoporotic compression fractures. Several trials have analyzed the effectiveness of vertebroplasty and kyphoplasty procedures although with discordant results (11–16).

One of the main criticisms advanced against traditional VAPs is the permanence of PMMA as a foreign body within the vertebral soma without any evidence of osteoinductive or reparative activity. The second critical aspect of PMMA is the risk of displacement and extravasation (leakage), which is the most frequent complication, especially in osteoporotic fractures (17).

Although bone cement displacement is a rare complication, some scholars believed that it could cause vertebral collapse, local instability of the spine, and pseudojoint formation, which may lead to intractable pain, aggravation of kyphosis, and even neurological impairment (18).

Leakage of cement from the vertebral body is relatively frequent and can occur either through bony continuum solutions or through veins departing from the vertebral soma. Cement leakage can affect the vertebral canal, leading to neurological problems. Extravasation into venous structures can lead to the phenomenon of embolism, especially at the pulmonary level, with severe consequences until death (19–21).

The use of a titanium microsphere system can plug cement leakage and simultaneously introduce, in the vertebral bone, consolidation material with documented biological activity.

Titanium microspheres are a filling material that offers immediate remedy for fracture stabilization and vertebral consolidation and at the same time is able to induce and initiate bone healing in the treatment of compression fractures. This aspect may represent a new concept and method in the vertebral body augmentation procedure.

The spherical-shaped trabecular structure, made using EBM—electron-beam melting technology, offers a scaffold for cell colonization mimicking the structure of the cancellous bone. Porous trabecular titanium, in fact, shows *in vitro*-marked osteoinductive and osteoconductive activity (22). The rough surface of 3D-printed EBM titanium trabeculae is prone to induce mesenchymal stem cell colonization and osteoblastic differentiation with bone matrix production (23), as demonstrated by electron microscopy, increased alkaline phosphatase activity, calcium deposition, and finally, a marked increase in the expression of genes typical of osteoblastic activity (24). Also, an *in vivo* study with 18-NAF PET documented the rapid induction of osteoblastic activity in the vertebral endplate by cages for interbody fusion built with the same trabecular titanium.

Porous microspheres are placed inside the vertebral soma after kyphoplasty balloon dilation, and they tend to form a stable cast with little likelihood of migration outside the created cavity and the surrounding bony gaps. This aspect may avoid the risk of embolism and neurological deficits related to cement overflow and leakage.

This system creates a compact agglomerate (inside the vertebrae) related to the “Velcro” effect generated between the trabeculae, guaranteeing stability to dynamic loads. A combination of the structure and the material used for production makes it possible to sustain the loads envisaged, with an elasticity module very similar to that of the bone tissue. The trabecular structure of the porous titanium spheres is similar to the spongy bone structure. It will be interesting to evaluate whether kyphoplasty with microspheres can permanently reduce and restore segmental kyphosis post-VCF (25).

**Figure 4:**
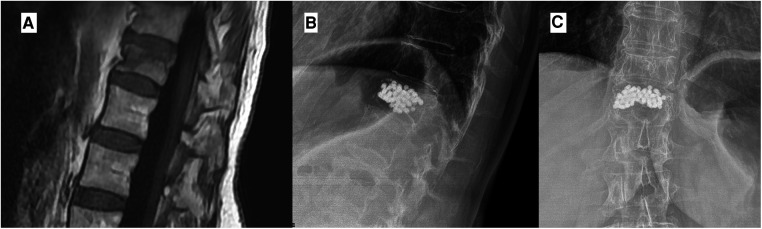
In this case, a 76-year-old woman with vertebral compression active fracture visible in MRI with STIR sequences (**A**) was treated with D11 vertebroplasty with the injection of titanium microspheres. The follow-up radiographic control shows the stability of the implant without any sign of leakage (**B,C**).

The osteoinductive capacity of the spheres can lead to a complete rehabilitation of the trabecular structure by osteoblastic cell and integration with the surrounding bone tissue. This aspect is likely related to the stability of the microsphere cast and fracture at 3 months’ follow-up (Figure 4).

An evaluation of the use of this type of material for “vertebral augmentation” may lead to a potential increase in the safety and efficacy of kyphoplasty procedures. The learning curve for this innovative system is not very steep since the instrumentation, introducer, and trokar are quite similar to those normally used for PMMA injection. Nevertheless, we suggest prospective studies to validate our preliminary results and suggestions.

## Further studies and limitations

The main limitation of this study was the low number of patients included in the sample, which did not allow us to assess statistical significance. In addition, it was not possible at this time to make an assessment of the recovery of lordosis loss or curvature [measurement of SL and others (22)], since some patients with very high VAS scores were not found suitable for preoperative standing imaging. We suggest that a larger study with a prospective arm can provide further validity for the introduction of alternate methods for the use of PMMA in VAPs. The protocol of the prospective observational study has been submitted on the Clinicaltrials.gov platform and is now in the stage of patient recruitment.

## Conclusion

This is the first clinical report describing the treatment of osteoporotic fractures by kyphoplasty with porous titanium microspheres. The procedure can be performed feasibly and stably using titanium microspheres with the goal of promoting better osteointegration and avoiding the risks of material leakage.

## Data Availability

The original contributions presented in the study are included in the article/Supplementary Material, and further inquiries can be directed to the corresponding author.
